# Deciding with the machine: hybrid intelligence and the future of clinical judgment in nursing

**DOI:** 10.1590/1518-8345.0000.5129

**Published:** 2026-10-09

**Authors:** Ricardo Limongi

**Affiliations:** 1 Universidade Federal de Goiás, Faculdade de Administração, Ciências Contábeis e Ciências Econômicas, Goiânia, GO, Brazil. ricardolimongi@ufg.br

**Figure:**
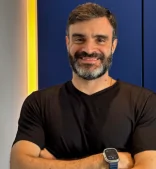

Artificial intelligence (AI) is no longer a distant promise in healthcare services: it operates, silently, at the bedside. Predictive scores of clinical deterioration, automated sepsis alerts, patient prioritization systems, and electronic health records with embedded suggestions have become part of the everyday infrastructure of care^1^. Nursing occupies a unique position in this landscape, as it is the profession that interacts most with these systems, receives the majority of their signals, and responds to them most frequently; paradoxically, it is also the least involved in their design and evaluation^2^. The debate over *whether* AI will enter clinical practice has concluded; the debate over *how* it should collaborate with professional judgment has barely begun. This editorial proposes a shift in this conversation: from algorithmic accuracy to the architecture of collaboration between nurses and intelligent systems.

## The problem is not the algorithm: it is cognition under automation

The customary rationale for adopting intelligent systems is to reduce the cognitive load on professionals who make decisions under pressure. Cumulative evidence, however, reveals a more ambiguous picture: poorly designed automation does not eliminate error - it displaces it.

Consider two recognizable scenes in any Latin American hospital. In the first, an inpatient ward nurse receives, on the same night, the third sepsis risk alert for patients whom, upon clinical examination, she considers stable. After dozens of false positives accumulated over weeks, the alert converts into noise, and the click that dismisses it becomes a reflex - a phenomenon known as alert fatigue. In the second scene, a novice professional postpones communicating the subtle deterioration of a patient because “the system did not flag anything”. This is automation bias: the documented tendency to over-rely on algorithmic recommendations, committing errors of commission (following incorrect advice) and omission (failing to act when the system remains silent)^3^.

Both scenes are two sides of the same coin. In one, an excess of signals erodes trust and induces automatic dismissal; in the other, the perceived authority of the system atrophies trained clinical vigilance. In both cases, the weakest link is not the predictive capacity of the model, but the encounter between system architecture and the real limits of human cognition-finite attention, heuristics, and time pressure. Evaluating only the accuracy of the algorithm, as most studies do, means measuring the wrong half of the problem.

## Hybrid intelligence as a design principle

The response to this impasse lies neither in further automation nor in resistance, but in a concept that has been gaining prominence in the literature on human-machine collaboration: hybrid intelligence - the deliberate combination of the complementary strengths of human and artificial intelligence, such that the collective outcome surpasses what either entity could achieve in isolation^4^.

The decisive word in this definition is deliberate. Complementarity is not an automatic byproduct of technological adoption; it is a property that must be intentionally constructed. In nursing practice, three design principles warrant emphasis. First, systems must calibrate trust rather than merely issue verdicts: an alert that displays why it was triggered, which variables were involved, and with what degree of uncertainty enables the professional to exercise judgment rather than blindly obey or disregard it. Second, dissent must be treated as data, not as a deviation: when a nurse disagrees with a score and records her assessment, this divergence should feed back into the system and institutional governance, converting clinical experience into organizational learning. Third, professional agency must be preserved by design: systems that demand bureaucratic justification for every disagreement penalize clinical judgment and practically demonstrate that it is less costly to agree with the machine.

Let us reimagine, under these principles, the previous scenes. The same night-shift nurse observes, alongside the alert, the triggering parameters and the model’s estimated confidence level; she disagrees, logs it with a single click, and proceeds to the bedside that *her own* expertise - not the system’s - identified as high priority. Months later, the pattern of dissents within that unit informs the recalibration of the model. Human judgment and algorithmic processing cease to compete for the final word and instead constitute a learning system.

## An agenda for Latin American nursing

This shift from the algorithm to collaboration opens an agenda across three fronts. For nursing research, it entails assuming leadership in studies that evaluate not only the accuracy of systems, but also the outcomes of the professional-algorithm dyad: when collaboration surpasses each component individually, under what conditions it fails, and how fatigue, expertise, and organizational context moderate this relationship. For education, it involves preparing nurses to *decide with* intelligent systems - incorporating algorithmic literacy, but above all, explicitly legitimizing reasoned dissent as a core clinical competency rather than technological insubordination. For management and regulation, it requires demanding transparency, continuous monitoring, and meaningful human oversight from deployed systems, in alignment with international principles of ethical AI governance in healthcare, which place human autonomy and accountability at the center^5^.

Finally, an ethical dimension spans all three fronts: when a hybrid decision fails, who bears responsibility? As long as liability rests entirely on the professional who “should have verified”, without systems providing real conditions for verification, the asymmetry will remain not only technical, but moral. Designing effective collaboration also entails ensuring a just distribution of responsibility.

Latin America, with its strained and unequal health systems, cannot afford to import technologies designed for other contexts and discover their side effects in practice. The region’s nursing workforce - numerous, pervasive, and present where no algorithm will ever be - possesses the epistemic authority to lead this conversation. Ultimately, the relevant question is not whether the algorithm is accurate, but whether the system was designed to listen to those standing at the patient’s bedside.
