# Supplementary material for: Deciding with the machine: hybrid intelligence and the future of clinical judgment in nursing
Source: Rev Lat Am Enfermagem. 2026 Oct 9;34:e5129. doi: 10.1590/1518-8345.0000.5129 (PMC13652528; doi:10.1590/1518-8345.0000.5129)
Supplement: Translation

# Translation: Decidir com a máquina: inteligência híbrida e o futuro do julgamento clínico em enfermagem

**DOI:** 10.1590/1518-8345.0000.5130

**Authors:** Ricardo Limongi

**Affiliations:** 1 Universidade Federal de Goiás, Faculdade de Administração, Ciências Contábeis e Ciências Econômicas, Goiânia, GO, Brasil. ricardolimongi@ufg.br

A inteligência artificial (IA) já não é uma promessa distante nos serviços de saúde: opera, silenciosamente, na beira do leito. Escores preditivos de deterioração clínica, alertas automatizados de sepse, sistemas de priorização de pacientes e prontuários eletrônicos com sugestões incorporadas tornaram-se parte da infraestrutura cotidiana do cuidado^1^. A enfermagem ocupa posição singular nesse cenário por ser a profissão que mais interage com esses sistemas, mais recebe seus sinais e mais responde e, paradoxalmente, é a que menos participa de seu desenho e avaliação^2^. O debate sobre *se* a IA entrará na prática clínica encerrou-se; o debate sobre *como* deve colaborar com o julgamento profissional mal começou. Este editorial propõe um deslocamento nessa conversa: da acurácia dos algoritmos para a arquitetura da colaboração entre enfermeiros e sistemas inteligentes.

## O problema não é o algoritmo: é a cognição sob automação

A justificativa habitual para adotar sistemas inteligentes é reduzir a carga cognitiva de profissionais que decidem sob pressão. A evidência acumulada, contudo, revela um quadro mais ambíguo: a automação mal desenhada não elimina o erro - desloca-o.

Considere duas cenas reconhecíveis em qualquer hospital latino-americano. Na primeira, uma enfermeira de unidade de internação recebe, na mesma noite, o terceiro alerta de risco de sepse para pacientes que, ao exame clínico, considera estáveis. Após dezenas de falsos positivos acumulados ao longo de semanas, o alerta converte-se em ruído, e o clique que o dispensa se torna reflexo, fenômeno conhecido como fadiga de alertas. Na segunda cena, um profissional recém-formado adia a comunicação da piora sutil de um paciente porque “o sistema não apontou nada”. É o viés de automação: a tendência documentada de sobreconfiar na recomendação algorítmica, cometendo erros de comissão (seguir conselhos incorretos) e de omissão (deixar de agir quando o sistema silencia)^3^.

As duas cenas são faces da mesma moeda. Em uma, o excesso de sinais corrói a confiança e induz à dispensa automática; na outra, a autoridade percebida do sistema atrofia a vigilância clínica treinada. Em ambas, o elo mais frágil não é a capacidade preditiva do modelo, mas o encontro entre a arquitetura do sistema e os limites reais da cognição humana, atenção finita, heurísticas, pressão de tempo. Avaliar apenas a acurácia do algoritmo, como faz a maioria dos estudos, é medir a metade errada do problema.

## Inteligência híbrida como princípio de desenho

A resposta a esse impasse não está em mais automação, tampouco em resistência, mas em um conceito que vem ganhando destaque na literatura sobre colaboração humano-máquina: a inteligência híbrida, a combinação deliberada das forças complementares da inteligência humana e da artificial, de modo que o conjunto supere o que cada parte alcançaria isoladamente^4^.

A palavra decisiva nessa definição é deliberada. A complementaridade não é subproduto automático da adoção tecnológica; é uma propriedade que precisa ser intencionalmente construída. Na prática da enfermagem, três princípios de desenho merecem destaque. Primeiro, os sistemas devem calibrar a confiança, e não apenas emitir veredictos: um alerta que exibe *por que* sinalizou quais variáveis, com que grau de incerteza, permite ao profissional exercer julgamento em vez de apenas obedecer ou ignorar. Segundo, o dissenso deve ser tratado como dado, não como desvio: quando a enfermeira discorda do escore e registra sua avaliação, essa divergência deveria realimentar o sistema e a governança institucional, convertendo a experiência clínica em aprendizado organizacional. Terceiro, a agência profissional deve ser preservada por desenho: sistemas que exigem justificativa burocrática para cada discordância punem o julgamento clínico e ensinam, na prática, que é mais barato concordar com a máquina.

Reimaginemos, sob esses princípios, as cenas anteriores. A mesma enfermeira da madrugada vê, junto ao alerta, os sinais que o dispararam e a confiança estimada do modelo; discorda, registra em um clique e segue para o leito que *seu* olhar - não o do sistema - identificou como prioritário. Meses depois, o padrão de dissensos daquela unidade orienta a recalibração do modelo. O julgamento humano e o processamento algorítmico deixam de competir pela última palavra e passam a compor um sistema que aprende.

## Uma agenda para a enfermagem latino-americana

Esse deslocamento, do algoritmo para a colaboração, abre uma agenda em três frentes. Para a pesquisa em enfermagem, trata-se de assumir protagonismo em estudos que avaliem não apenas a acurácia dos sistemas, mas os desfechos da díade profissional-algoritmo: quando a colaboração supera cada parte isolada, sob quais condições fracassa, e como fadiga, expertise e contexto organizacional moderam essa relação. Para a formação, trata-se de preparar enfermeiros para *decidir com* sistemas inteligentes, o que inclui alfabetização algorítmica mas sobretudo a legitimação explícita do dissenso fundamentado como competência clínica, não como insubordinação tecnológica. Para a gestão e a regulação, trata-se de exigir dos sistemas implantados transparência, monitoramento contínuo e supervisão humana significativa, em consonância com os princípios internacionais de governança ética da IA em saúde, que colocam a autonomia humana e a responsabilização no centro^5^.

Há, por fim, uma dimensão ética que atravessa as três frentes: quando a decisão híbrida erra, quem responde? Enquanto a responsabilidade recair integralmente sobre o profissional que “deveria ter verificado”, sem que os sistemas lhe ofereçam condições reais de verificação, a assimetria será não apenas técnica, mas moral. Desenhar boa colaboração é, também, distribuir justamente a responsabilidade.

A América Latina, com seus sistemas de saúde pressionados e desiguais, não pode dar-se ao luxo de importar tecnologias desenhadas para outros contextos e descobrir seus efeitos colaterais na prática. A enfermagem da região, numerosa, capilar, presente onde o algoritmo jamais estará, tem autoridade epistêmica para liderar essa conversa. No fim, a pergunta que importa não é se o algoritmo acerta, mas se o sistema foi desenhado para ouvir quem está ao lado do paciente.

# Translation: Decidir con la máquina: inteligencia híbrida y el futuro del juicio clínico en enfermeira

**DOI:** 10.1590/1518-8345.0000.5128

**Authors:** Ricardo Limongi

**Affiliations:** 1 Universidade Federal de Goiás, Faculdade de Administração, Ciências Contábeis e Ciências Econômicas, Goiânia, GO, Brasil. ricardolimongi@ufg.br

La inteligencia artificial (IA) ya no es una promesa distante en los servicios de salud: opera, silenciosamente, junto al lecho del paciente. Las escalas predictivas de deterioro clínico, las alertas automatizadas de sepsis, los sistemas de priorización de pacientes y las historias clínicas electrónicas con sugerencias integradas se han convertido en parte de la infraestructura cotidiana del cuidado^1^. La enfermería ocupa una posición singular en este escenario por ser la profesión que más interactúa con estos sistemas, más señales recibe de ellos y más responde ante las mismas; paradójicamente, es la que menos participa en su diseño y evaluación^2^. El debate sobre *si* la IA ingresará a la práctica clínica ha concluido; el debate sobre *cómo* debe colaborar con el juicio profesional apenas comienza. Este editorial propone un desplazamiento en esta conversación: de la exactitud de los algoritmos a la arquitectura de la colaboración entre las enfermeras y los sistemas inteligentes.

## El problema no es el algoritmo: es la cognición bajo automatización

La justificación habitual para la adopción de sistemas inteligentes es reducir la carga cognitiva de los profesionales que toman decisiones bajo presión. Sin embargo, la evidencia acumulada revela un panorama más ambiguo: la automatización mal diseñada no elimina el error, sino que lo desplaza.

Considere dos escenas reconocibles en cualquier hospital latinoamericano. En la primera, una enfermera de una unidad de hospitalización recibe, en la misma noche, la tercera alerta de riesgo de sepsis para pacientes a quienes, al examen clínico, considera estables. Tras docenas de falsos positivos acumulados a lo largo de semanas, la alerta se convierte en ruido, y el clic que la descarta se vuelve un reflejo, fenómeno conocido como fatiga de alertas. En la segunda escena, un profesional recién graduado pospone la comunicación sobre el deterioro sutil de un paciente porque «el sistema no indicó nada». Es el sesgo de automatización: la tendencia documentada a confiar en exceso en la recomendación algorítmica, incurriendo en errores de comisión (seguir consejos incorrectos) y de omisión (dejar de actuar cuando el sistema permanece en silencio)^3^.

Ambas escenas constituyen las dos caras de una misma moneda. En una, el exceso de señales erosiona la confianza e induce al descarte automático; en la otra, la autoridad percibida del sistema atrofia la vigilancia clínica capacitada. En ambos casos, el eslabón más débil no es la capacidad predictiva del modelo, sino el encuentro entre la arquitectura del sistema y los límites reales de la cognición humana: atención finita, heurísticas y presión de tiempo. Evaluar únicamente la precisión del algoritmo, como realiza la mayoría de los estudios, equivale a medir la mitad equivocada del problema.

## Inteligencia híbrida como principio de diseño

La respuesta a este dilema no radica en una mayor automatización ni en la resistencia, sino en un concepto que viene cobrando relevancia en la literatura sobre colaboración humano-máquina: la inteligencia híbrida, es decir, la combinación deliberada de las fortalezas complementarias de la inteligencia humana y la artificial, de tal modo que el conjunto supere lo que cada parte lograría de manera aislada^4^.

La palabra clave en esta definición es deliberada. La complementariedad no es un subproducto automático de la adopción tecnológica; es una propiedad que debe construirse intencionalmente. En la práctica de enfermería, destacan tres principios de diseño. En primer lugar, los sistemas deben calibrar la confianza y no limitarse a emitir veredictos: una alerta que expone por qué se activó, qué variables consideró y con qué grado de incertidumbre, permite al profesional ejercer su juicio en lugar de limitarse a obedecer o ignorar. En segundo lugar, la discrepancia debe tratarse como un dato y no como una desviación: cuando la enfermera disiente de la puntuación y registra su valoración, esa divergencia debería retroalimentar el sistema y la gobernanza institucional, convirtiendo la experiencia clínica en aprendizaje organizacional. En tercer lugar, la autonomía profesional debe preservarse desde el diseño: los sistemas que exigen una justificación burocrática para cada desacuerdo penalizan el juicio clínico y enseñan, en la práctica, que resulta menos costoso asentir ante la máquina.

Reimaginemos, bajo estos principios, las escenas anteriores. La misma enfermera del turno nocturno observa, junto a la alerta, los parámetros que la activaron y el nivel de confianza estimado del modelo; no está de acuerdo, lo registra con un clic y se dirige a la cama del paciente que *su propio* criterio - no el del sistema - identificó como prioritario. Meses después, el patrón de discrepancias de esa unidad orienta la recalibración del modelo. El juicio humano y el procesamiento algorítmico dejan de competir por la última palabra y pasan a integrar un sistema que aprende.

## Una agenda para la enfermería latinoamericana

Este desplazamiento, del algoritmo a la colaboración, plantea una agenda en tres frentes. Para la investigación en enfermería, se trata de asumir un rol protagónico en estudios que evalúen no solo la exactitud de los sistemas, sino los resultados de la díada profesional-algoritmo: cuándo la colaboración supera a cada parte de forma aislada, bajo qué condiciones fracasa y de qué manera la fatiga, la experiencia y el contexto organizacional moderan esta relación. Para la formación académica, implica preparar a las enfermeras para *decidir junto* a sistemas inteligentes, lo cual incluye la alfabetización algorítmica, pero, sobre todo, la legitimación explícita del disenso fundamentado como una competencia clínica y no como insubordinación tecnológica. Para la gestión y la regulación, exige demandar transparencia, monitoreo continuo y una supervisión humana significativa de los sistemas implantados, en consonancia con los principios internacionales de gobernanza ética de la IA en salud, los cuales ubican la autonomía humana y la rendición de cuentas en el centro^5^.

Existe, finalmente, una dimensión ética que atraviesa los tres frentes: cuando una decisión híbrida falla, ¿quién asume la responsabilidad? Mientras la responsabilidad recaiga íntegramente en el profesional que “debió haber verificado”, sin que los sistemas le ofrezcan condiciones reales para dicha verificación, la asimetría no solo será técnica, sino también moral. Diseñar una colaboración adecuada implica, asimismo, distribuir la responsabilidad de manera justa.

América Latina, con sus sistemas de salud presionados y desiguales, no puede permitirse el lujo de importar tecnologías diseñadas para otros contextos y descubrir sus efectos colaterales en la práctica. La enfermería de la región - numerosa, capilar y presente allí donde el algoritmo jamás estará - posee la autoridad epistémica para liderar este debate. En última instancia, la pregunta fundamental no es si el algoritmo acierta, sino si el sistema fue diseñado para escuchar a quien se encuentra al lado del paciente.
